# An MDM2 inhibitor achieves synergistic cytotoxic effects with adenoviruses lacking *E1B55kDa* gene on mesothelioma with the wild-type *p53* through augmenting NFI expression

**DOI:** 10.1038/s41419-021-03934-y

**Published:** 2021-07-02

**Authors:** Thao Thi Thanh Nguyen, Masato Shingyoji, Michiko Hanazono, Boya Zhong, Takao Morinaga, Yuji Tada, Hideaki Shimada, Kenzo Hiroshima, Masatoshi Tagawa

**Affiliations:** 1 Division of Pathology and Cell Therapy, Chiba Cancer Center Research Institute, 666-2 Nitona, Chuo-ku, Chiba 260-8717 Japan; 2 Department of Molecular Biology and Oncology, Graduate School of Medicine, Chiba University, 1-8-1 Inohana, Chuo-ku, Chiba 260-8670 Japan; 3 Division of Medical Biotechnology, Biotechnology Center of Ho Chi Minh City, 2374 National Highway 1, District 12, Ho Chi Minh, Vietnam; 4 Division of Respirology, Chiba Cancer Center, 666-2 Nitona, Chuo-ku, Chiba 260-8717 Japan; 5 Department of Biochemistry and Genetics, Graduate School of Medicine, Chiba University, 1-8-1 Inohana, Chuo-ku, Chiba 260-8670 Japan; 6 Department of Respirology, Graduate School of Medicine, Chiba University, 1-8-1 Inohana, Chuo-ku©, Chiba 260-8670 Japan; 7 Department of Respiratory Medicine, International University of Health and Welfare Atami Hospital, 13-1 Higasikaigan, Atami, 413-0012 Japan; 8 Department of Surgery, Graduate School of Medicine, Toho University, 6-11-1 Oomori-nishi, Oota-ku, 143-8541 Tokyo Japan; 9 Department of Pathology, Tokyo Women’s Medical University Yachiyo Medical Center, 477-96 Ohwadashinden, Yachiyo, 276-8524 Japan; 10 Funabashi Orthopedic Hospital, 1-833 Hazama, Funabashi, 274-0822 Japan

**Keywords:** Cancer, Molecular biology

## Abstract

A majority of mesothelioma specimens were defective of p14 and p16 expression due to deletion of the INK4A/ARF region, and the p53 pathway was consequently inactivated by elevated MDM2 functions which facilitated p53 degradaton. We investigated a role of p53 elevation by MDM2 inhibitors, nutlin-3a and RG7112, in cytotoxicity of replication-competent adenoviruses (Ad) lacking the p53-binding *E1B55kDa* gene (Ad-delE1B). We found that a growth inhibition by p53-activating Ad-delE1B was irrelevant to p53 expression in the infected cells, but combination of Ad-delE1B and the MDM2 inhibitor produced synergistic inhibitory effects on mesothelioma with the wild-type but not mutated *p53* genotype. The combination augmented p53 phosphorylation, activated apoptotic but not autophagic pathway, and enhanced DNA damage signals through ATM-Chk2 phosphorylation. The MDM2 inhibitors facilitated production of the Ad progenies through augmented expression of nuclear factor I (NFI), one of the transcriptional factors involved in Ad replications. Knocking down of p53 with siRNA did not increase the progeny production or the NFI expression. We also demonstrated anti-tumor effects by the combination of Ad-delE1B and the MDM2 inhibitors in an orthotopic animal model. These data collectively indicated that upregulation of wild-type p53 expression contributed to cytotoxicity by E1B55kDa-defective replicative Ad through NFI induction and suggested that replication-competent Ad together with augmented p53 levels was a therapeutic strategy for *p53* wild-type mesothelioma.

## Introduction

Mutations of *p53* gene were infrequent in mesothelioma but a majority of the clinical specimens did not express p14 or p16 due to either deletion of the encoding CDKN2A locus or methylation of the transcriptional regulatory regions [1]. Recent genome-wide studies also confirmed the CDKN2A deletion and loss of p53 functions in the majority of mesothelioma despite having the wild-type *p53* genotype [2, 3]. The loss of p14 expression resulted in enhanced MDM2 activity, which mediated ubiquitination and subsequent degradation of p53. The lack of p16 led to activation of cyclin-dependent kinase 4/6, which increased pRb phosphorylation and promoted cell cycle progression. Most of the mesothelioma are therefore defective of these tumor suppressor functions. The characteristic genetic alterations suggested that p53 re-expression played a crucial role in a therapeutic strategy for mesothelioma [4]. In fact forced expression of p53 in mesothelioma not only activated p53-mediated cell death pathways but dephosphorylated pRb since p21, one of the p53 targets, blocked cyclin-dependent kinase 2 [5]. These data collectively indicated that the p53 downstream pathways were intact in mesothelioma and that restoration of p53 expression was the therapeutic options.

Replication-competent adenoviruses (Ad) induced cytotoxicity preferentially in tumors and were tested for the clinical feasibility [6]. Nevertheless, a role of p53 in the Ad-mediated cytotoxicity was not well understood. Upregulated p53 levels facilitated death of Ad-infected cells and then decreased production of the cytotoxic virus progenies [7, 8], whereas elevated p53 promoted tumor cell death [9]. Ad replication by itself influenced endogenous p53 expression in the infected cells. E1A molecules increased a p53 level, but E1B55kDa inactivated p53 [10]. We therefore examined effects of Ad defective of the gene encoding E1B55kDa molecules (Ad-delE1B) on mesothelioma with the wild-type *p53* genotype, and demonstrated that Ad-delE1B upregulated p53 expression and produced synergistic cytotoxicity with cisplatin [11]. These data suggested that increased p53 expression was favorable for cytotoxicity induced by Ad and a DNA damaging agent.

Expression of p53 is regulated not only at the transcriptional level but in the ubiquitination process. Ubiquitinated p53 is subjected to proteasome-mediated degradation and MDM2 plays a major role in the process. An agent to inhibit the MDM2 function can consequently increase endogenous p53 levels. An imidazole compound such as nutlin-3a and RG7112 functions as an MDM2 inhibitor, upregulated p53 levels and activated p53-mediated apoptosis in tumors with the wild-type *p53* genotype [12, 13].

In this study, we examined how p53 upregulation by Ad-delE1B contributed to the Ad-mediated cytotoxicity, and investigated effects of MDM2 inhibitors on Ad-delE1B-mediated cell death and the viral replications. The present study demonstrated a role of p53 in activation of DNA damage signals and a cellular factor involved in Ad replications.

## Materials and methods

### Cells, agents, and mice

Mesothelioma with the wild-type *p53*, MSTO-211H, NCI-H226, and NCI-H28 cells, were obtained from ATCC, and those with mutated *p53*, EHMES-1 and JMN-1B cells, were from Dr. Hamada (Hiroshima University, Japan), and A549 lung carcinoma cells were from Cell Resource Center for Biomedical Research (Sendai, Japan). All the p53 wild-type mesothelioma cells were defective of the CDKN2A locus. Nutlin-3a was obtained from Selleck (Houston, TX), and RG7112 from Roche TCRC (New York, NY) or MedChem (Monmouth Junction, NJ). BALB/c nu/nu mice were purchased from SLC (Hamamatsu, Japan). Cells were negative for mycoplasma and authorized by STR analysis.

### Ad preparations

Replication-competent Ad-delE1B, Ad-p53 or Ad expressing β-galactosidase gene (Ad-LacZ) were prepared with an Adeno-X expression system (Takara, Shiga, Japan). Virus particles (vp) were estimated with the formula: absorbance at 260 nm in 0.1% sodium dodecyl sulfate × 1.1 × 10^12^.

### Western blot analysis

Cell lysate was subjected to electrophoresis, transferred onto a nylon filter and hybridized with antibody (Ab) against phosphorylated p53 at Ser 15, p21, cleaved capase-3, poly-(ADP ribose) polymerase (PARP) (that also detects cleaved PARP), cleaved caspase-8 at Asp381, Fas, FADD, cleaved caspase-9, Bax, PUMA, Atg-5, Beclin-1, LC3A/B, Chk2, ATR, phosphorylated Chk1 at Ser 345, phosphorylated Chk2 at Thr68, H2AX, actin (Cell Signaling, Danvers, MA), MDM2, E1A, Chk1, B23, TFIID, NFI (Santa Cruz, Dallas, TX), Oct1, ATM (Millipore, Temecula, CA), phosphorylated ATM at Ser 1981, KAP1, phosphorylated KAP1 at Ser 824 (Abcam, Cambridge, UK), phosphorylated H2AX at Ser 139 (γ-H2AX) (BioLegend, San Diego, CA), p53, phosphorylated ATR at Ser 428 (Thermo Fisher, Fremont, CA).

### RNA interference and virus production

Cells were transfected with siRNA targeting p53 (#TP53-VHS40367) or non-specific siRNA (#12935-114) (Thermo Fisher) as a control using Lipofectamine RNAiMAX according to the manufacturer’s protocol (Thermo Fisher). For detecting viral production, cells were treated with Ad-delE1B and/or MDM2 inhibitors, and the lysate was examined for cytotoxicity with A549 cells. The virus titers were calculated with the TCID_50_ method.

### Cell viability assay

Cells were treated with Ad-delE1B for 4 days or MDM2 inhibitors for 2 days. For combination treatments, cells were infected with Ad-delE1B for 2 days and then treated with MDM2 inhibitors for further 2 days. Cell viability was assayed with a cell-counting WST kit (Wako, Osaka, Japan), (WST assay). Combinatory effects were examined by CalcuSyn software (Biosoft, Cambridge, UK). Combination index (CI) values at respective fraction affected (Fa) points which showed relative levels of suppressed cell viability were calculated based on the WST assay. CI < 1, CI = 1 and CI > 1 indicate synergistic, additive, and antagonistic actions, respectively. Viable cell numbers were counted with the trypan blue dye exclusion test (Wako).

### Cell cycle analysis and apoptosis

Cells treated with RNase and propidium iodide (PI) (50 μg/ml) were analyzed for the cell cycle with FACSCalibur (BD, San Diego, CA). Cells were also stained with annexin V Alexa Fluor 488 and PI, and analyzed for apoptosis with an image-based cytometer (Invitrogen, Carlsbad, CA).

### Immunofluorescence

Cells treated with 4% paraformaldehyde, 0.25% Triton X-100 and 5% bovine serum albumin were incubated with anti-p53 or anti-NFI Ab and secondary Ab conjugated with Alexa Fluor 488 or Alexa Fluor 555. Nuclei were stained with 4′,6-diamidine-2-phenylindole (DAPI) (1 µg/ml) or PI (50 µg/ml). The images were analyzed with a laser confocal microscope (Leica, Deerfield, IL).

### Animal studies

All animal experiments have been performed according to the guideline of Chiba University and were permitted from the animal experiment committee (permission number: A2-160 and A2-161). Four-week-old BALB/c nu/nu mice were purchased from SLC (Hamamatsu, Japan). All mice were housed in clean conditions with soft food and water in the animal resource center at Chiba University. These mice were randomly divided into eight groups (6 mice/group). BALB/c nude mice were anesthetized with pentobarbital sodium (1%, 0.01 ml/g) (Sigma-Aldrich, Darmstadt, Germany) before injected with MSTO-211H cells (5 × 10^6^ cells) into the pleural cavity on day 1, treated with an intrapleural injection of Ad on day 4, and with an intraperitoneal injection of MDM2 inhibitors on day 10. Mice were measured body weight every 2 days. Mice were sacrificed by cervical dislocation after being anesthetized on day 25. Xenografts were harvested and tumor weights were measured.

### Statistical analyses

All data are presented as the mean ± SE. One-way analysis of variance followed by post hoc Tukey’s test was used to assess significant differences between two groups or more. *p* value <0.05 was considered statistically significant. Statistical analyses were performed using GraphPad Prism 6 software (GraphPad software, La Jolla, CA, USA).

## Results

### Ad-delE1B induced p53-independent growth suppression

**Fig. 1:**
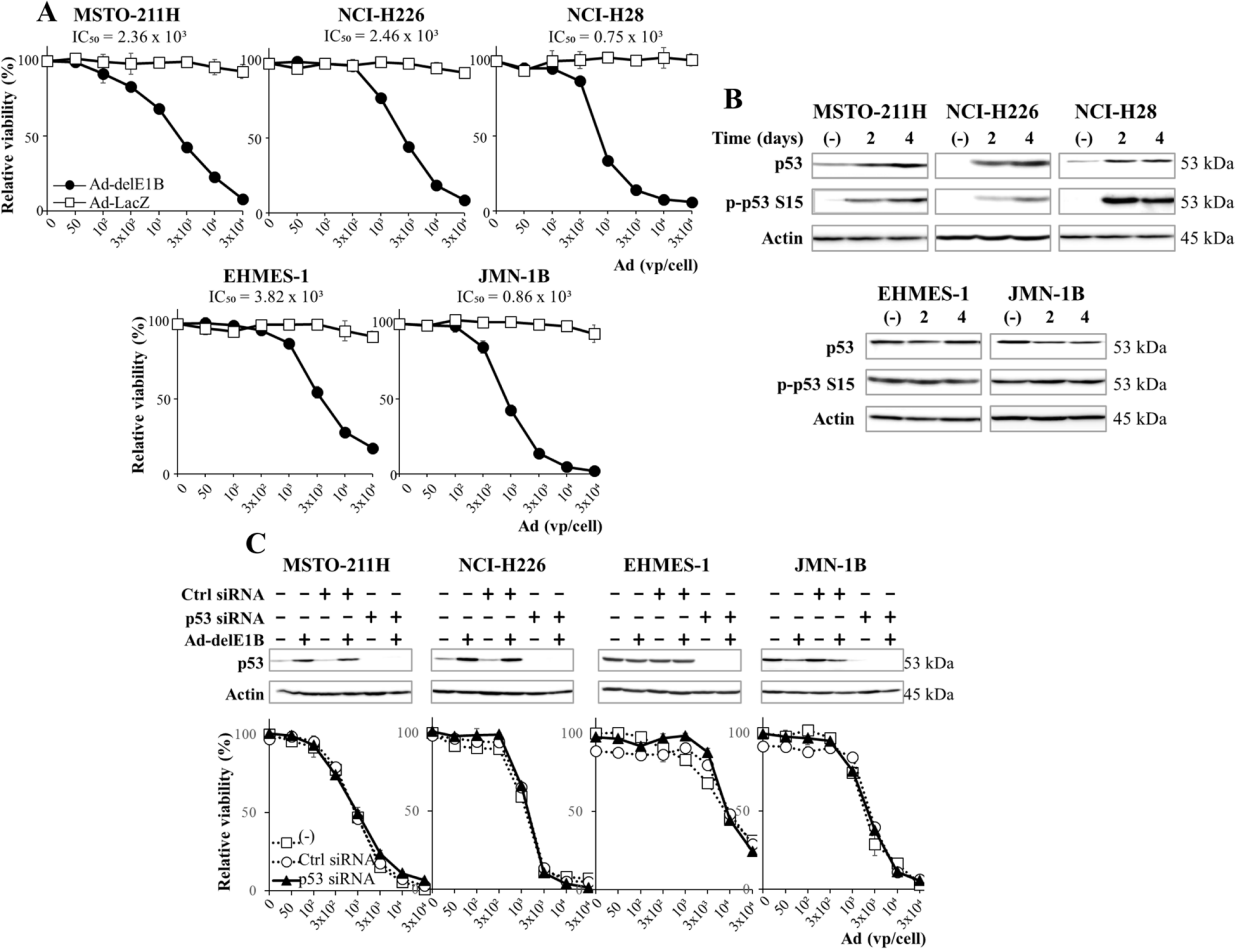
Ad-delE1B-mediated growth inhibitory effects were independent of p53 expression. **A** Growth inhibitory effects of Ad-delE1B. Mesothelioma with the wild-type *p53* (MSTO-211H, NCI-H226 and NCI-H28) or mutated *p53* genotype (EHMES-1 and JMN-1B) were treated with Ad-delE1B or Ad-LacZ at various vp for 4 days. The cell viabilities were measured with the WST agent and relative viability was calculated based on untreated cells. IC_50_ values (vp/cell) were calculated with GraphPad Prism software. Averages and SE bars are shown (*n* = 3). **B** Cells were treated with Ad-delE1B (3 × 10^3^ vp/cell) and cultured for 2 or 4 days. Expression of p53, phosphorylated p53 at Ser 15 (p-P53 S15), and actin as a control was examined with Western blot analysis. **C** Cells transfected with p53 siRNA or with non-target control siRNA (Ctrl siRNA) were treated with Ad-delE1B (3 × 10^3^ vp/cell) for 4 days. Expression of p53 and actin as a control was examined with Western blot analysis. Cells transfected with siRNA were also treated with Ad-delE1B at various vp for 4 days and the cell viabilities were measured with the WST agent. Relative viability was calculated based on untreated cells. Averages and SE bars are shown (*n* = 3).

We investigated growth inhibitory effects of Ad-delE1B on mesothelioma with wild-type (MSTO-211H, NCI-H226 and NCI-H28) or mutated *p53* genotype (EHMES-1 and JMN-1B) (Fig. 1A). All cells were susceptible to Ad-delE1B but not to Ad-LacZ as a control. These cells showed similar IC_50_ for inhibition, suggesting that Ad-delE1B-mediated effects were irrelevant to the *p53* genotype. We also examined p53 expression and the phosphorylation by Ad-delE1B (Fig. 1B). The expression and phosphorylation at Ser 15 were upregulated in the wild-type *p53* cells, whereas those in mutated cells remained unchanged or decreased. These data indicated that Ad-delE1B increased endogenous wild-type p53 levels but the upregulated expression did not contribute to growth inhibition.

We examined a role of p53 in the Ad-delE1B-induced growth suppression by knocking down p53 (Fig. 1C). Cells treated with p53 siRNA eliminated p53 expression irrespective of *p53* genotypes and the p53 levels remained suppressed even after Ad-delE1B infection. Susceptibility of p53 siRNA-treated cells to Ad-delE1B was not different from that of cells untreated or treated with control siRNA regardless of the *p53* genotype. These data indicated that Ad-delE1B-induced cytotoxicity was independent of p53 levels and increased p53 expression by Ad-delE1B did not influence the cytotoxicity.

### MDM2 inhibitors-mediated growth suppression

**Fig. 2:**
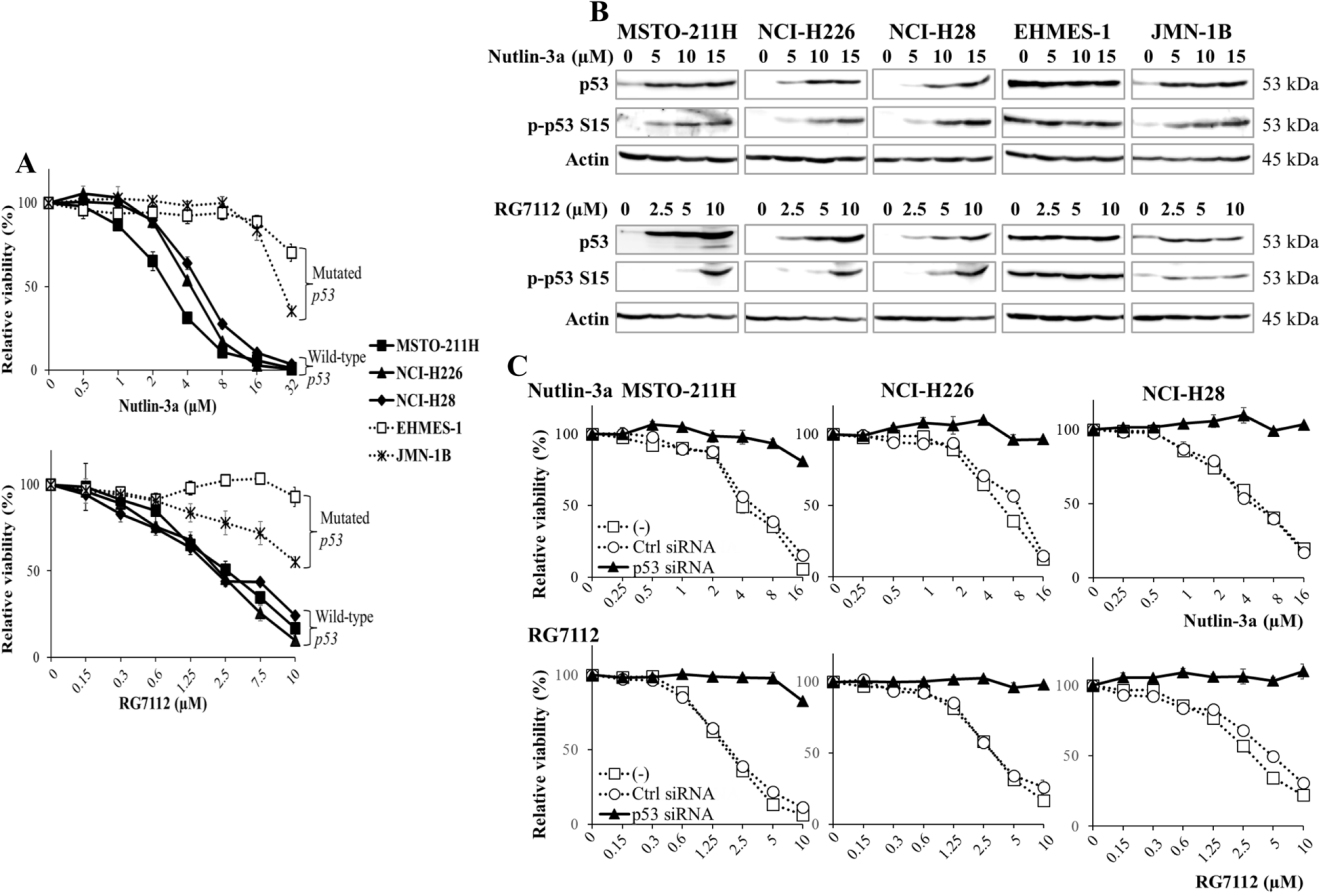
MDM2 inhibitors increased p*53* expression in wild-type p53 mesothelioma and the growth inhibition was relevant to p*53* genotype. **A** Cells were treated with various concentrations of nutlin-3a for 4 days or RG7112 for 3 days. The cell viabilities were measured with the WST agent and relative viability was calculated based on untreated cells. Averages and SE bars are shown (*n* = 3). **B** Cells were treated with various concentrations of nutlin-3a or RG7112 as indicated for 2 days. Expression of p53 and phosphorylated p53 at Ser 15, and actin as a control was examined with western blot analysis. **C** Cells transfected with p53 siRNA or control siRNA were treated with various concentrations of nutlin-3a or RG7112 for 3 days and the cell viabilities were measured with the WST agent. Relative viability was calculated based on untreated cells. Averages and SE bars are shown (*n* = 3).

We examined susceptibility of mesothelioma to MDM2 inhibitors, nutlin-3a and RG7112 (Fig. 2A, Supplementary Table 1). Both inhibitors achieved inhibitory effects to mesothelioma with wild-type greater than those with mutated *p53* genotype. Average IC_50_ values of nutlin-3a to wild-type *p53* cells (4.07 ± 0.59 μM) was lower than those to mutated *p53* cells (30.85 ± 2.76) (*p* < 0.01). Likewise, the values of RG7112 in wild-type cells (2.55 ± 0.43) were less than those in mutated cells (10.51 ± 0.49) (*p* < 0.01). We then examined the expression of p53 and the phosphorylation in cells treated with MDM2 inhibitors (Fig. 2B). Wild-type *p53* cells augmented p53 and the phosphorylation levels in a dose-dependent manner. We observed similar changes in JMN-1B cells despite being insensitive the inhibitors. EHMES-1 cells did not show increase of p53 or the phosphorylation. We further examined a role of p53 in the MDM2 inhibitors-induced cytotoxicity by knocking down p53 (Fig. 2C). All the wild-type *p53* cells became insensitive to nutlin-3a or RG7112 after p53 siRNA treatments, whereas cells treated with control siRNA remained sensitive. These data indicated that MDM2 inhibitors produced cytotoxicity in a p53-dependent manner through increased endogenous p53 levels.

### Ad-delE1B and MDM2 inhibitors produced combinatory effects

**Fig. 3:**
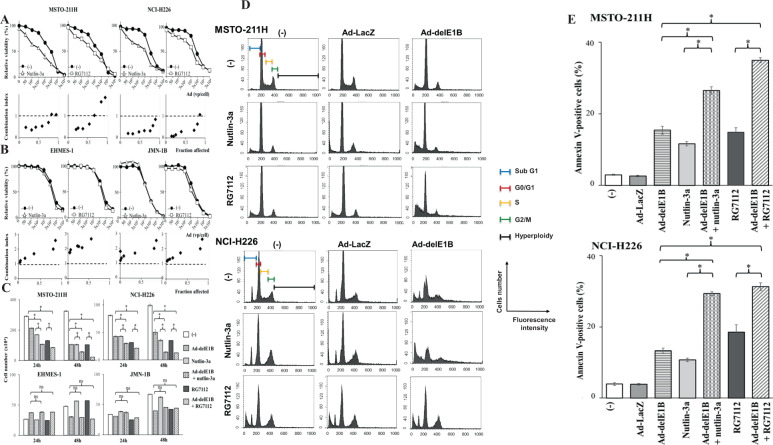
MDM2 inhibitors produced synergistic growth inhibitory effects and changed cell cycle progression with Ad-delE1B on wild-type p*53* cells. **A** Wild-type *p53* or **B** mutated *p53* cells were infected with various vp of Ad-delE1B for 2 days, and then treated with nutlin-3a (MSTO-211H: 0.5 µM, NCI-H226, EHMES-1, and JMN-1B: 1 µM) or RG7112 (0.15 µM) for further 2 days. The cell viabilities were measured with the WST agent. Relative viability was calculated based on untreated cells. Averages and SE bars are shown (*n* = 3). CI values were plotted at respective Fa points. CI < 1, CI = 1 and CI > 1 indicate synergistic, additive, and antagonistic actions, respectively. **C** Cells were infected with Ad-delE1B (MSTO-211H and NCI-H226: 1.5 × 10^3^ vp/cell, EHMES-1 and JMN-1B: 3 × 10^3^ vp/cell) for 2 days, and then treated with nutlin-3a (MSTO-211H and NCI-H226: 5 µM, EHMES-1, and JMN-1B: 15 µM) or RG7112 (MSTO-211H and NCI-H226: 3 µM, EHMES-1 and JMN-1B: 10 µM). Live cells numbers were then counted on 24 and 48 h later. ns not significant, *p < 0.001 (GraphPad Prism 6, La Jolla). **D** Cells were infected with Ad-delE1B or Ad-LacZ (3 × 10^3^ vp/cell) for 2 days, and then treated with nutlin-3a (5 µM) or RG7112 (5 µM). Cell cycle distribution was determined with flow cytometry. Representative histogram of MSTO-211H cells at 36 h after treatment with the inhibitor and that of NCI-H226 at 48 h. **E** Percentages of annexin V-positive cells. Cells were treated as above and annexin V-positive cells were determined with flow cytometry in MSTO-211H cells at 36 h after the inhibitor treatment and in NCI-H226 at 48 h. Data are presented as averages with SE bars (*n* = 3). *p < 0.01.

We investigated a role of upregulated p53 in Ad-delE1B-mediated cytotoxicity with MDM2 inhibitors. We treated Ad-delE1B-infected cells with the inhibitors and examined growth inhibition (Fig. 3A). Combination of Ad-delE1B and nutlin-3a or RG7112 showed CI values less than 1 at Fa points between 0.2 and 0.8 (nutlin-3a) or between 0.2 and 0.7 (RG7112) in MSTO-211H and NCI-H226 cells, demonstrating that the combination produced synergistic effects. A combinatory use of Ad-delE1B and MDM2 inhibitors however produced rather antagonistic effects in EHMES-1 and JMN-1B cells because the CI values were above 1 (Fig. 3B). We also examined the combinatory effects on cell survivals with a dye exclusion assay (Fig. 3C). Ad-delE1B or MDM2 inhibitors alone produced cytotoxicity in wild-type *p53* mesothelioma and the combination achieved cytotoxic effects greater than a single treatment. In contrast, EHMES-1 and JMN-1B cell numbers decreased with Ad-delE1B at 48 h but no further inhibition was detected in the combination. These results suggested that combination of Ad-delE1B and MDM2 inhibitors achieved growth inhibition in a p53-dependent manner.

### Augmented apoptosis by the combination

We examined cell cycle progression and apoptosis induction of the wild-type *p53* cells treated with the combination (Fig. 3D, Supplementary Table 2). MDM2 inhibitors decreased S-phase populations and Ad-delE1B induced hyperploidy (over 4N) fractions in MSTO-211H cells. The combination increased sub-G1 and hyperplioidy compared with cells treated with Ad-delE1B alone. Likewise, MDM2 inhibitors decreased S-phase populations, and Ad-delE1B increased sub-G1 and hyperploidy fractions in NCI-H226 cells which constantly showed a different peak in a sub-G1 region from cell death-associated sub-G1 fractions. The combination increased sub-G1 populations greater than the single treatment and augmented hyperploidy. In contrast, Ad-LacZ did not influence cell cycle and combination of Ad-LacZ and the inhibitors showed similar cell cycle distributions as the inhibitor alone-treated case. These data indicated that MDM2 inhibitors enhanced Ad-delE1B-mediated effects.

We also examined apoptosis induction with the annexin V staining (Fig. 3E). An annexin V-positive fraction increased in cells infected with Ad-delE1B or treated with MDM2 inhibitors. The combination expanded the population greater than the single treatment in MSTO-211H and NCI-H226 cells. These data showed that the combination augmented apoptotic cell death.

### Combinations augmented p53 and caspase cleavages

**Fig. 4:**
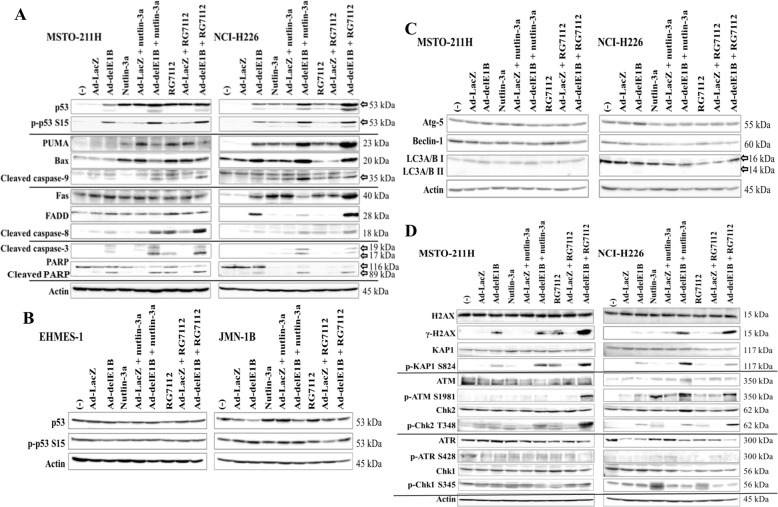
Combination of Ad-delE1B and MDM2 inhibitors augmented apoptotic pathways and DNA damage signals. **A** Cells were infected with Ad-delE1B or Ad-LacZ (3 × 10^3^ vp/cell) for 2 days, and then treated with nutlin-3a (15 µM) or RG7112 (10 µM) for further 16 h in MSTO-211H or 36 h in NCI-H226. Cell lysate of wild-type (**A**) or mutated *p53* cells (**B**) were subjected to western blot analysis and expression levels of molecules associated with p53 (**A**, **B**), apoptosis (**A**), autophagy (**C**), and DNA damage pathways (**D**) were examined with respective Ab as indicated.

We investigated the expression of p53 and the related molecules in wild-type *p53* cells treated with the combination (Fig. 4A). Cells treated with Ad-delE1B, nutlin-3a, or RG7112 showed increased p53 levels and the phosphorylation, and those with the combination further augmented the expression. Ad-delE1B induced cleavage of caspase-3 and PARP, and the combination further induced the cleavages. The combination increased caspase-8 cleavage caused by Ad-delE1B although expression of Fas and FADD was not enhanced in the combination in MSTO-211H cells. In contrast, NCI-H226 cells showed differential expression levels of Fas and FADD in the combination. Fas expression was upregulated in NCI-226H cells treated with Ad-delE1B or the inhibitors, and the FADD increased in those with Ad-delE1B. Combination with RG7112 enhanced Fas and FADD expression, whereas the combination with nultin-3a rather decreased the levels. Ad-delE1B and the inhibitors stimulated cleavage of caspase-9 at 35 kDa molecules, and the combination further augmented the cleavages in both cells. Expression of PUMA and Bax increased in MSTO-211H cells treated with either nutlin-3a or RG7112 but decreased in those with the combination. In contrast, the levels in NCI-226H cells treated with the combination were greater than those treated with a single agent. Transduction with Ad-LacZ scarcely influenced these expressions even in the combination except increased PUMA levels in nutlin-3a-treated MSTO-211H cells. Expression of p53 and the phosphorylation remained unchanged in EHMES-1 cells and was not augmented in JMN-1B cells treated with the combination (Fig. 4B). These data suggested that the combination augmented p53 levels and activated apoptotic pathways in wild-type *p53* mesothelioma but activation of the extrinsic or intrinsic pathways was dependent on cells tested.

We also examined whether autophagy pathways were relevant to cell death (Fig. 4C). Expression of Atg-5, Beclin-1, or transition of LC3A/B I to LC3A/B II was scarcely influenced by Ad-delE1B or MDM2 inhibitor, and was not enhanced by the combination. These data indicated that autophagy was not induced in wild-type *p53* cells.

### Combination activated DNA damage signals

We examined DNA damage responses induced by Ad-delE1B and the combination (Fig. 4D). Expression of γ-H2AX increased with Ad-delE1B and to a lesser extent with MDM2 inhibitors, and the level was further augmented in the combination. Phosphorylated KAP1 at Ser 824, a different DNA damage marker, was induced by Ad-delE1B and RG7112, and was further elevated in the combination. We also analyzed the expression of molecules in ATM-Chk2 and ATR-Chk1 pathways which were relevant to H2AX and KAP1 phosphorylation. Expression levels of ATM, Chk1 and Chk2 were constant among cells treated with Ad-delE1B and/or MDM2 inhibitors, but phosphorylation of ATM at Ser 1981 and Chk2 at Thr 348 was augmented in MSTO-211H cells treated with combination of Ad-delE1B and RG7112. Ad-delE1B, nutlin-3a or RG7112 alone increased the phosphorylation of ATM and Chk2 in NCI-H226 cells, and the combination further stimulated the phosphorylation. ATR expression remained unchanged in MSTO-211H cells but that in NCI-H226 cells was down-regulated by Ad infections and the combination. Phosphorylation levels of ATR at Ser 428 and Chk1 at Ser 345 remained unchanged in cells treated with the agent alone or the combination except elevated Chk1 phosphorylation in NCI-H226 cells treated with nutlin-3a or RG7112. These results indicated that Ad-delE1B in combination with MDM2 inhibitors-induced DNA damage mainly through activation of the ATM-Chk2 rather than the ATR-Chk1 pathway.

### MDM2 inhibitors augmented Ad-delE1B replications and suppressed tumor growth in vivo

**Fig. 5:**
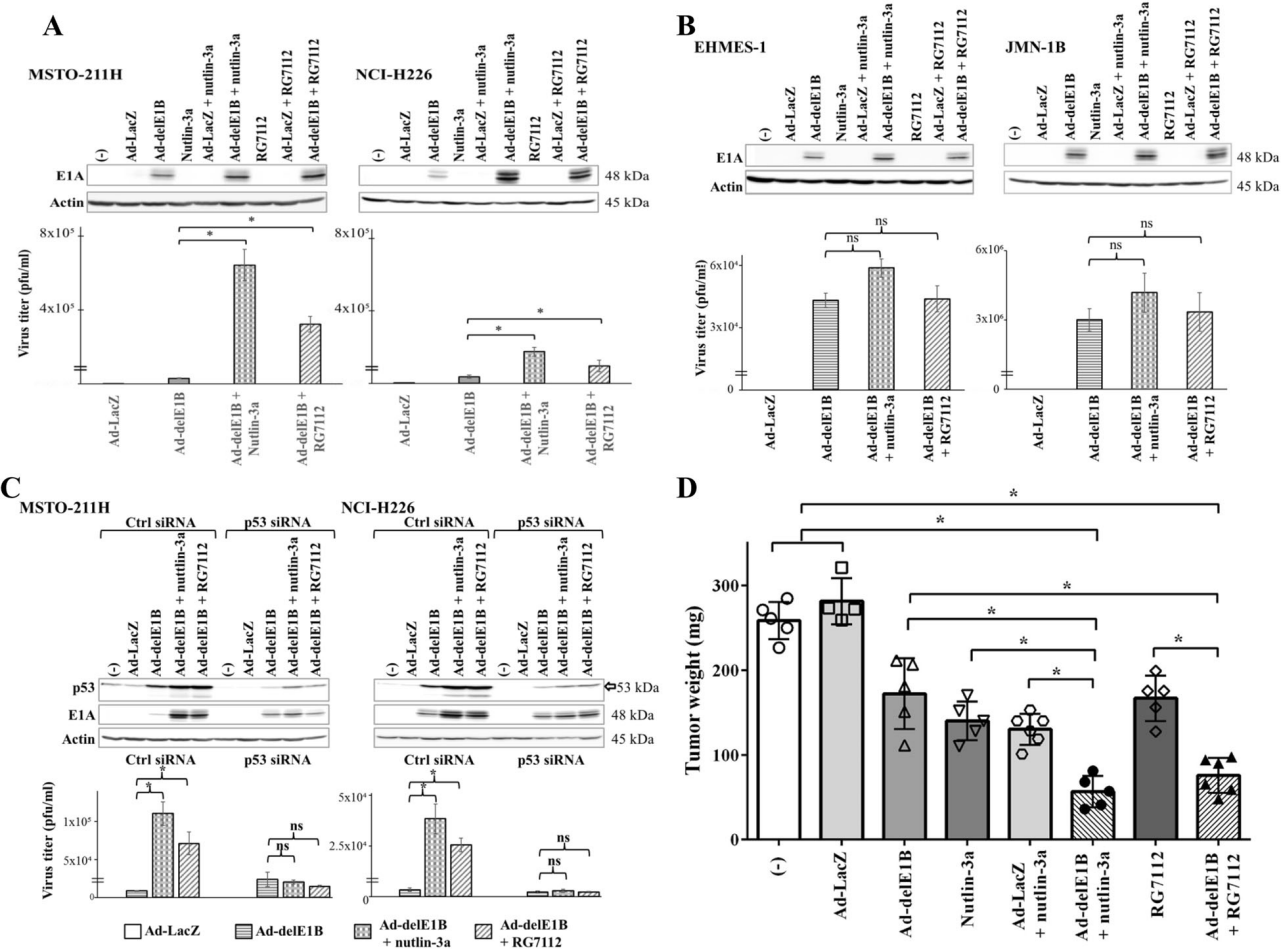
MDM2 inhibitors augmented production of Ad-delE1B progenies through p53 expression and combination suppressed tumor growth in vivo. Wild-type (**A**, **C**) and mutated *p53* cells (**B**) were infected with Ad-delE1B or Ad-LacZ (3 × 10^3^ vp/cell) for 2 days, and treated with nutlin-3a (15 µM) or RG7112 (10 µM) for further 16 h in MSTO-211H or 36 h in NCI-H226. Expression of p53, E1A, and actin as a control was examined with Western blot analysis. Quantitation of virus progenies was examined with the TCID_50_ method. Averages and SE bars are shown (*n* = 3). ns not significant, *p < 0.01. **C** Expression of p53 was knocked down with p53 siRNA and control siRNA was used as a control. Cells transfected with siRNA were infected with Ad-delE1B for 2 days, and then treated with nutlin-3a (15µM) or RG7112 (10 µM) for further 16 h in MSTO-211H or 36 h in NCI-H226. **D** Ad-delE1B (1 × 10^10^ vp/mouse) and MDM2 inhibitors (1 mg) produced combinatory anti-tumor effects in an animal experiment. Ad-LacZ was used as a control. The data indicated individual tumor weights and an interquartile range of weights of tumors. *p < 0.01.

We investigated a role of MDM2 inhibitors in production of viral progenies (Fig. 5). Ad-delE1B-infected cells expressed E1A, a product of the immediate early gene, and the expression was upregulated in combination of MDM2 inhibitors (Fig. 5A). We estimated the progeny numbers with the TCID_50_ method and found that both inhibitors enhanced the production in the wild-type *p53* cells. In contrast, mutated *p53* mesothelioma did not increase the production with MDM2 inhibitors and the E1A expression remained unchanged (Fig. 5B). We further examined an involvement of p53 in enhancing viral replications by knocking down p53 (Fig. 5C). Cells treated with p53 siRNA downregulated Ad-delE1B-induced p53 levels and minimally augmented the levels with MDM2 inhibitors. Ad-delE1B-induced EIA was not influenced by the siRNA treatment but the MDM2 inhibitors-mediated E1A upregulation decreased. A titration of viral productions indicated that enhanced Ad-delE1B replications by the inhibitors were eliminated with the p53 siRNA and the production was indifferent between cells infected with Ad-delE1B and those treated with the combination. These data demonstrated that an increased p53 level by MDM2 inhibitors facilitated replications of Ad-delE1B.

We next evaluated anti-tumor effects of the combination in a xenograft model (Fig. 5D). An administration of Ad-delE1B, nutlin-3a, or RG7112 alone inhibited tumor growth and the combination further reduced the tumor weights. No significant weight loss was observed in mice treated with the combination. These in vivo data showed that the combination produced combinatory anti-tumor effects on mesothelioma developed in the pleura cavity. We also confirmed that Ad-delE1B-mediated E1A expression of tumors was upregulated by a combinatory use of MDM2 inhibitors (Supplementary Fig. 4).

### Enhanced NFI expression by MDM2 inhibitors

**Fig. 6:**
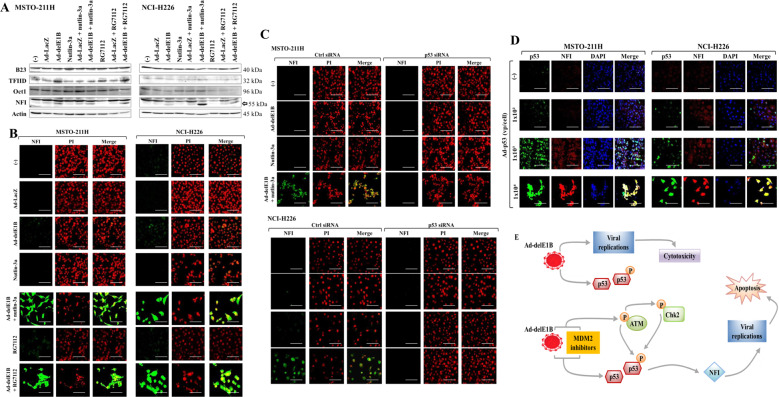
Enhanced NFI expression with MDM2 inhibitors. Cells were infected with Ad-delE1B or Ad-LacZ (3 × 10^3^ vp/cell) for 2 days, and then treated with nutlin-3a (15 µM) or RG7112 (10 µM) for further 16 h in MSTO-211H or 36 h in NCI-H226. **A** Cell lysate was examined for expression of cellular factors linked with Ad replications with Western blot analysis. NFI: An upper band ubiquitously expressed was non-specific and a lower band corresponding to 55 kDa was authentic. **B**–**D** Cells were stained with Ab against NFI or p53, or with DAPI or PI, and the images were analyzed with a laser confocal microscope. **C** Expression of p53 was knocked down with p53 siRNA and non-target siRNA was used as a control. Cells transfected with siRNA were infected with Ad-delE1B for 2 days, and then treated with nutlin-3a (15 µM) or RG7112 (10 µM) for further 16 h in MSTO-211H or 36 h in NCI-H226. **D** Cells were infected with several vp of Ad-p53 as indicated for 2 days. **E** A model of Ad-delE1B and p53 upregulated by MDM2 inhibitors in Ad-mediated cytotoxicity and virus replications. Scale bar: 10 μm.

We analyzed the expression of four major transcriptional factors, B23, TFIID, Oct1, and NFI, involved in Ad replications (Fig. 6A). Expression of B23 was not influenced by Ad-delE1B, MDM2 inhibitors, or the combinations. TFIID were augmented in MSTO-211H cells treated with Ad-delE1B and the combination with RG7112, but remained unchanged in NCI-H226 cells. Oct1 expression was downregulated in NCI-H226 cells treated with RG7112 and combination of Ad-delE1B and RG7112, but was not affected in MSTO-211H cells. In contrast, NFI expression increased with Ad-delE1B infections, and was further augmented in the combination with nutlin-3a in NCI-H226 cells. The combination with RG7112 also enhanced NFI expression in both cells. We also examined the upregulated NFI levels with immunostaining data (Fig. 6B). MSTO-211H and NCI-H226 cells infected with Ad-delE1B but not Ad-LacZ-induced NFI, but those treated with nutlin-3a or RG7112 remained negative for NFI. Combination of Ad-delE1B and the inhibitors strongly augmented NFI expression in the nucleus and cytoplasm. These data collectively suggested that Ad-delE1B-induced NFI expression was mediated by upregulated p53 expression.

We then investigated a role of p53 in NFI expression with siRNA (Fig. 6C) and replication-deficient Ad expressing wild-type p53 (Ad-p53) (Fig. 6D). MSTO-211H and NCI-H226 cells treated with p53 siRNA did not express NFI when they were infected with Ad-delE1B or treated with the combinatory use of nutlin-3a. Transduction of Ad-p53 augmented p53 levels and induced NFI expression according to Ad amounts. We noticed that Ad-p53 mainly induced NFI in nuclei in contrast to cells treated with the combination. These data indicated that induction of NFI was associated with p53 expression and NFI was one of the p53 target molecules.

## Discussion

We investigated a role of p53 in cytotoxicity induced by p53-activating replication-competent Ad and demonstrated that increase of endogenous p53 with MDM2 inhibitors augmented Ad-induced DNA damage responses and promoted the viral replications through augmented NFI expression. Ad-delE1B upregulated p53 levels in the wild-type *p53* mesothelioma but the p53 upregulation did not contribute to the Ad-mediated cytotoxicity. Nevertheless, further augmentation of p53 levels with an MDM2 inhibitor produced synergistic cytotoxicity in combination with Ad-delE1B. In addition, the present study firstly demonstrated to our knowledge that the p53 level played a vital role in NFI induction and subsequent production of Ad progenies.

Ad-delE1B preferentially replicated in tumors and immortalized cells, but not in normal cells (Supplementary Fig. 1). The replications were irrelevant to the *p53* genotype of infected cells [14]. We previously reported that Ad-delE1B increased p53 levels and induced apoptosis in wild-type *p53* mesothelioma with CDKN2A deletion, and suggested a role of p53 in Ad-delE1B-induced cytotoxicity [11]. Moreover, forced expression of p14 impaired the Ad-delE1B-mediated cytotoxicity [15] and consequently MDM2 activity was also involved in the cytotoxicity. The current study further demonstrated that the cytotoxicity was not linked with p53 expression and combination with MDM2 inhibitors increased the Ad-delE1B cytotoxicity. These data therefore suggested a putative threshold level of p53 as for Ad-mediated cytotoxicity. Previous studies investigated a role of p53 in cytotoxicity of replication-competent Ad and demonstrated that p53 enhanced the cytotoxicity [16]. The Ad used in these studies were different in the structures from Ad-delE1B, for example, expressing mutated p53 which was degradation-resistant but functional in the transcriptional activity [17], or having an expression unit of wild-type p53 [18, 19]. A possible mechanism for the enhanced cytotoxicity was not well analyzed in these studies but several mechanisms were postulated depending on Ad structures, which included autophagy induction [18], microRNA-induced p21 inhibition [19], and p53-mediated apoptosis [6]. Viral replications examined in these reports were scarcely influenced or rather decreased due to facilitated death of viruses-producing cells [6, 9, 11]. Furthermore, they used tumors in which the p53 downstream pathways were disturbed [6, 18]. We presumed that NFI was not well induced in these p53 non-functional cells and a non-NFI mechanism was consequently involved in such cases. A possible p53 role in cytotoxicity of replication-competent Ad can be influenced by cellular factors, which achieve different outcomes as for viral progeny productions.

We demonstrated that nutlin-3a and RG7112 increased endogenous p53 levels in combination with Ad-delE1B and induced cleavages of caspase-3, -8 and -9, and PARP. The combination however differentially influenced expression levels of Fas, FADD, Bax, and PUMA. Expression of Fas and FADD for example remained unchanged in MSTO-211H cells treated with Ad-delE1B and nutlin-3a or RG7112, whereas that in NCI-H226 cells was differently affected. These data indicated that upregulated p53 levels activated caspase cleavages in wild-type *p53* cells but detailed mechanisms of apoptosis induction were not identical among cells tested. Nevertheless, the combination invariably augmented Ad-delE1B-induced DNA damage responses, which were evidenced by increased γ-H2AX and phosphorylated KAP1 levels. ATM and Chk2 were involved in the DNA damage induced by Ad-delE1B [20] and we demonstrated that an MDM2 inhibitor enhanced the ATM-Chk2-mediated DNA damage, but the ATR-Chk1 pathway was scarcely involved. The differential DNA damage responses suggested that Ad-delE1B replications induced double-strand DNA breaks and MDM2 inhibitors preferentially supported ATM-Chk2 activation which was linked with p53 phosphorylation. KAP1 was phosphorylated by Ad replications as well as a non-viral mechanism, and the phosphorylation was further upregulated by E1B55kDa deletion since KAP1 bound to E1B55kDa molecules [20, 21]. KAP1 is in fact a negative regulator for Ad replications but the phosphorylation of KAP1 promoted decondensation of cellular and viral genome, which resulted in transcriptional activation of the genes required for Ad replications. The increased KAP1 phosphorylation by MDM2 inhibitors was therefore attributable to enhanced DNA damage induced by viral replications and also contributed to production of viral progenies. We also noticed increased hyperploidy fractions induced by Ad-delE1 and the combination with MDM2 inhibitors. We previously showed that the hyperploidy was not linked with aberrant chromosomal numbers or abnormal cell divisions [11] and speculated that the hyperploidy may be partly associated with increased nuclear DNA contents caused by Ad replications.

We demonstrated that MDM2 inhibitors enhanced viral production in the wild-type *p53* cells and knocking down of p53 decreased the progeny production to the level of Ad-infected cells, which indicated that p53 levels regulated the viral replications. The viral production in normal cells with intact CDKN2A region is interesting but Ad-delE1B were less cytotoxic (Supplementary Fig. 1) and MDM2 inhibitors were only toxic at a high concentration in normal cells. A possible effect of the combination on normal tissues can be examined in vivo but we did not observe body weight loss in the animal study (data not shown). The current study also examined cellular factors involved in Ad replications and showed that NFI expression was slightly induced by Ad-delE1B and further upregulated by MDM2 inhibitors [22]. Knocking down of p53 depleted the NFI upregulation, and transduction with Ad-p53 increased the expression in a dose-dependent manner. These data indicated that the NFI expression level was regulated by p53. Induction of NFI in Ad-delE1B-infected cells was therefore attributable to increased endogenous p53, but we cannot rule out a possible induction of NFI by Ad replications. A compared use of Ad-delE1B and replication-competent Ad bearing p53-inactivating E1B55kDa molecules can differentiate contribution of p53 and viral replications to NFI induction. We also tested a role of NFI in the viral production by knocking down of NFI with siRNA and demonstrated that NFI regulated the production (Supplementary Fig. 2). We noticed that an expression level of NFI may not be directly associated with the production of the viral progenies because the production in MSTO-211H cells was greater than that in NCI-226H cells. These data suggested that the viral replications were influenced by many cellular factors including synthesis of viral late proteins and the viral packaging activities.

The present study firstly reported that p53 augmented NFI levels and demonstrated a possible p53-mediated upregulation of NFI. A previous study however showed p53-induced NF1 downregulation in hepatocellular carcinoma [23]. Moreover, NFI augmented p53 expression in mammary glands [24] and decreased the expression in glioblastoma [25]. These data therefore suggested that a reciprocal regulation between p53 and NFI and the regulation was dependent on cells used. We then examined the regulation by knocking down of NFI with siRNA in wild-type *p53* mesothelioma (Supplementary Fig. 3). We found that decreased NFI expression downregulated p53 levels, indicating that NFI was a positive regulator for p53. NFI is a transcriptional factor and regulates a number of genes [26], but how NFI controls the *p53* gene remains unclear. A putative binding site of NFI or p53 in the regulatory region of respective genes was not identified and consequently mechanism of the reciprocal regulation between NFI and p53 remains unknown. NFI showed a tumor-promoting activity through facilitating cell proliferations and migration [25], and consequently elevated NFI expression may be linked with preferential cytotoxicity of oncolytic Ad in tumors compared with normal tissues. A functional role of p53 and NFI in Ad-delE1B-mediated cytotoxicity can be different from that in other types of replication-competent Ad types bearing E1B55kDa molecules. E1B55kDa mediated KAP1 dephosphorylation and p53 degradation, and influenced a number of signal pathways [20, 27]. A possible axis of ATM-Chk2-p53-NFI demonstrated in the present study can be restricted in the Ad-delE1B-induced cytotoxicity. Moreover, NFI induction can be subjected to a putative p53 threshold level since Ad-delE1B minimally induced NFI expression in contrast to the significant induction in the combination with MDM2 inhibitors. On the other hand, we detected a dose-dependent NFI induction with Ad-p53, suggesting that the NFI level was correlated with p53 expression when the p53 level increased beyond the threshold.

We demonstrated a clinical feasibility of the combination in an in vivo experimental model. Ad-delE1B is clinically used in China [28] and p53-activating agents were examined for the efficacy in a clinical setting [29, 30]. Mesothelioma increased p53 levels through inhibition of MDM2 and DNA damage, and consequently activated p53 downstream pathway and cell death induction. These data suggest that p53 stimulation is a target of mesothelioma treatments. An injection of Ad vectors into the pleural cavity is technically easy and several clinical studies demonstrated that intrapleural administration of Ad was safe [31]. A clinical trial of the combination can reveal therapeutic efficacy and possible adverse effects on normal tissues as well.

In conclusions, we showed that increase of p53 levels by Ad-delE1B infection did not contribute to the Ad-mediated cytotoxicity, but further enhanced p53 expression with MDM2 inhibitors was associated with activation of the ATM-Chk2 pathway and augmented the cytotoxicity through upregulated NFI expression (summarized in Fig. 6E). A majority of mesothelioma has a characteristic genetic alteration regarding the p53 expression and the present study suggested that a combinatory use of Ad-delE1B and a p53-augmenting MDM2 inhibitor was a therapeutic strategy for mesothelioma with the wild-type *p53* genotype.

## Supplementary information

Supplementary Table 1

Supplementary Table 2

Supplementary Figure 1

Supplementary Figure 2

Supplementary Figure 3

Supplementary Figure 4
