# Supplementary material for: An MDM2 inhibitor achieves synergistic cytotoxic effects with adenoviruses lacking E1B55kDa gene on mesothelioma with the wild-type p53 through augmenting NFI expression
Source: Cell Death Dis. 2021 Jul 2;12(7):663. doi: 10.1038/s41419-021-03934-y (PMC8260618; doi:10.1038/s41419-021-03934-y)
Supplement: Supplementary file 1 — Supplementary Table 1 [file 41419_2021_3934_MOESM1_ESM.docx]

**Supplementary Table 1. IC_50_ values of MDM2 inhibitors to mesothelioma.**

|  |  | **IC_50_ (µM) ± SE** | |
| --- | --- | --- | --- |
| **Cells** | ***p53* genotype** | **Nutlin-3a** | **RG7112** |
| **MSTO-211H** | **wild-type** | **3.02 ± 0.83** | **3.36 ± 0.73** |
| **NCI-H226** | **wild-type** | **4.10 ± 0.94** | **2.40 ± 0.54** |
| **NCI-H28** | **wild-type** | **5.09 ± 0.82** | **1.89 ± 0.09** |
| **EHMES-1** | **mutated** | **33.61 ± 9.19** | **11.00 ± 2.03** |
| **JMN-1B** | **mutated** | **28.09 ± 6.02** | **10.01 ± 2.08** |

**IC_50_ values were calculated with GraphPad Prism software.**
