# Supplementary material for: An MDM2 inhibitor achieves synergistic cytotoxic effects with adenoviruses lacking E1B55kDa gene on mesothelioma with the wild-type p53 through augmenting NFI expression
Source: Cell Death Dis. 2021 Jul 2;12(7):663. doi: 10.1038/s41419-021-03934-y (PMC8260618; doi:10.1038/s41419-021-03934-y)
Supplement: Supplementary file 2 — Supplementary Table 2 [file 41419_2021_3934_MOESM2_ESM.docx]

**Supplementary** Table 2. Cell cycle changes caused by Ad-delE1B and/or MDM2 inhibitors

| Cells | Time | Treatment | Cell cycle distribution (%) (Average ± SE) | | | | | |
| --- | --- | --- | --- | --- | --- | --- | --- | --- |
|  | (hrs) |  | Sub-G1 | G0/G1 | S | G2/M | Hyperploidy | |
| MSTO-211H | 16 | (-) | 2.64 ± 0.09 | 53.02 ± 0.30 | 19.02 ± 0.27 | 24.34 ± 0.50 | 1.40 ± 0.06 |  |
|  |  | Ad-LacZ | 0.87 ± 0.06 | 80.49 ± 0.19 | 5.83 ± 0.10 | 11.67 ± 0.14 | 1.30 ± 0.03 |  |
|  |  | Ad-delE1B | 1.92 ± 0.06 | 74.77 ± 0.21 | 5.63 ± 0.03 | 13.29 ± 0.14 | 4.55 ± 0.14 |  |
|  |  | Nutlin-3a | 3.73 ± 0.01 | 84.62 ± 1.06 | 0.96 ± 0.04 | 9.08 ± 0.28 | 0.84 ± 0.05 |  |
|  |  | Ad-LacZ + nutlin-3a | 0.59 ± 0.06 | 84.22 ± 0.22 | 2.12 ± 0.04 | 12.82 ± 0.25 | 0.92 ± 0.02 |  |
|  |  | Ad-delE1B + nutlin-3a | 8.21 ± 0.25 | 70.07 ± 0.21 | 2.46 ± 0.08 | 12.83 ± 0.15 | 6.71 ± 0.23 |  |
|  |  | RG7112 | 5.45 ± 0.27 | 81.95 ± 0.14 | 1.40 ± 0.21 | 10.57 ± 0.30 | 0.73 ± 0.05 |  |
|  |  | Ad-LacZ + RG7112 | 1.25 ± 0.08 | 78.05 ± 0.12 | 0.62 ± 0.03 | 18.48 ± 0.15 | 1.60 ± 0.07 |  |
|  |  | Ad-delE1B + RG7112 | 9.57 ± 0.16 | 65.26 ± 0.75 | 2.82 ± 0.04 | 15.38 ± 0.39 | 7.22 ± 0.31 |  |
|  | 24 | (-) | 1.75 ± 0.07 | 54.65 ± 0.08 | 19.87 ± 0.21 | 23.91 ± 0.12 | 1.31 ± 0.08 |  |
|  |  | Ad-LacZ | 1.61 ± 0.06 | 84.62 ± 0.12 | 3.06 ± 0.01 | 9.69 ± 0.10 | 1.26 ± 0.06 |  |
|  |  | Ad-delE1B | 5.27 ± 0.07 | 73.68 ± 0.07 | 5.76 ± 0.07 | 9.80 ± 0.29 | 5.88 ± 0.10 |  |
|  |  | Nutlin-3a | 3.93 ± 0.02 | 86.44 ± 0.28 | 1.30 ± 0.06 | 7.59 ± 0.16 | 1.07 ± 0.09 |  |
|  |  | Ad-LacZ + nutlin-3a | 1.32 ± 0.06 | 84.13 ± 0.11 | 0.54 ± 0.06 | 13.06 ± 0.19 | 0.93 ±0.01 |  |
|  |  | Ad-delE1B + nutlin-3a | 24.10 ± 0.30 | 53.40 ± 0.16 | 4.51 ± 0.23 | 8.59 ± 0.16 | 10.05 ± 0.08 |  |
|  |  | RG7112 | 5.26 ± 0.10 | 83.01 ± 0.06 | 1.25 ± 0.08 | 9.62 ± 0.24 | 1.27 ± 0.10 |  |
|  |  | Ad-LacZ + RG7112 | 5.15 ± 0.19 | 75.86 ± 0.23 | 1.54 ± 0.15 | 16.00 ± 0.12 | 1.41 ± 0.03 |  |
|  |  | Ad-delE1B + RG7112 | 23.31 ± 0.35 | 50.77 ± 0.51 | 4.28 ± 0.08 | 12.20 ± 0.10 | 9.70 ± 0.10 |  |
| NCI-H226 | 24 | (-) | 10.55 ± 0.16 | 47.54 ± 0.11 | 13.95 ±0.24 | 25.57 ± 0.34 | 2.37 ± 0.13 | |
|  |  | Ad-LacZ | 11.91 ± 0,10 | 60.27 ± 0.06 | 9.78 ± 0.11 | 17.62 ± 0.15 | 1.48 ± 0.10 | |
|  |  | Ad-delE1B | 17.26 ± 0.16 | 38.50 ± 0.18 | 11.39 ± 0.09 | 16.69 ± 0.12 | 16.93 ± 0.30 | |
|  |  | Nutlin-3a | 9.34 ± 0.19 | 50.57 ± 0.36 | 3.57 ± 0.08 | 34.60 ± 0.57 | 2.48 ± 0.21 | |
|  |  | Ad-LacZ + nutlin-3a | 10.46 ± 0.24 | 57.57 ± 0.56 | 5.28 ± 0.08 | 25.56 ± 0.38 | 2.23 ± 0.07 | |
|  |  | Ad-delE1B + nutlin-3a | 26.00 ± 0.26 | 33.80 ± 0.28 | 7.26 ± 0.12 | 13.71 ± 0.15 | 19.95 ± 0.22 | |
|  |  | RG7112 | 13.30 ± 0.19 | 49.78 ± 0.16 | 4.44 ± 0.09 | 31.17 ± 0.41 | 2.00 ± 0.15 | |
|  |  | Ad-LacZ + RG7112 | 13.70 ± 0.04 | 55.68 ± 0.23 | 6.49 ± 0.27 | 24.11 ± 0.46 | 1.63 ± 0.13 | |
|  |  | Ad-delE1B + RG7112 | 29.01 ± 0.35 | 33.56 ± 0.21 | 7.73 ± 0.21 | 14.44 ± 0.16 | 16.15 ± 0.34 | |
|  | 48 | (-) | 12.07 ± 0.40 | 57.93 ± 0.95 | 11.09 ± 0.20 | 18.15 ± 1.75 | 1.33 ± 0.33 | |
|  |  | Ad-LacZ | 10.37 ± 0.04 | 65.66 ± 0.29 | 6.36 ± 0.14 | 16.6 ± 0.31 | 2.17 ± 0.03 | |
|  |  | Ad-delE1B | 22.96 ± 0.19 | 40.51 ± 0.39 | 11.31 ± 0.29 | 15.87 ± 0.26 | 10.65 ± 0.34 | |
|  |  | Nutlin-3a | 11.76 ± 0.33 | 52.78 ± 0.18 | 4.22 ± 0.13 | 29.71 ± 0.33 | 2.13 ± 0.12 | |
|  |  | Ad-LacZ + nutlin-3a | 10.20 ± 0.16 | 58.90 ± 0.06 | 4.57 ± 0.12 | 23.32 ± 0.11 | 3.58 ± 0.11 | |
|  |  | Ad-delE1B + nutlin-3a | 38.02 ± 0.39 | 26.17 ± 0.34 | 7.23 ± 0.10 | 11.49 ± 0.06 | 17.35 ± 0.15 | |
|  |  | RG7112 | 24.73 ± 0.61 | 47.18 ± 0.35 | 3.45 ± 0.25 | 22.79 ± 0.72 | 2.33 ± 0.11 | |
|  |  | Ad-LacZ + RG7112 | 28.30 ± 0.32 | 50.52 ± 0.22 | 4.93 ± 0.20 | 15.82 ± 0.11 | 1.64 ± 0.02 | |
|  |  | Ad-delE1B + RG7112 | 49.07 ± 0.24 | 22.83 ± 0.05 | 6.55 ± 0.15 | 8.74 ± 0.10 | 12.97 ± 0.12 | |

Cells were treated with Ad (3x10^3^ vp/cell) and/or nutlin-3a (5µM) or RG7112 (5µM) for 16-48 hrs as indicated and cell cycle progression was analyzed with flow cytometry.
