# Supplementary figures and images for: An MDM2 inhibitor achieves synergistic cytotoxic effects with adenoviruses lacking E1B55kDa gene on mesothelioma with the wild-type p53 through augmenting NFI expression
Source: Cell Death Dis. 2021 Jul 2;12(7):663. doi: 10.1038/s41419-021-03934-y (PMC8260618; doi:10.1038/s41419-021-03934-y)

Supplementary Figure 1

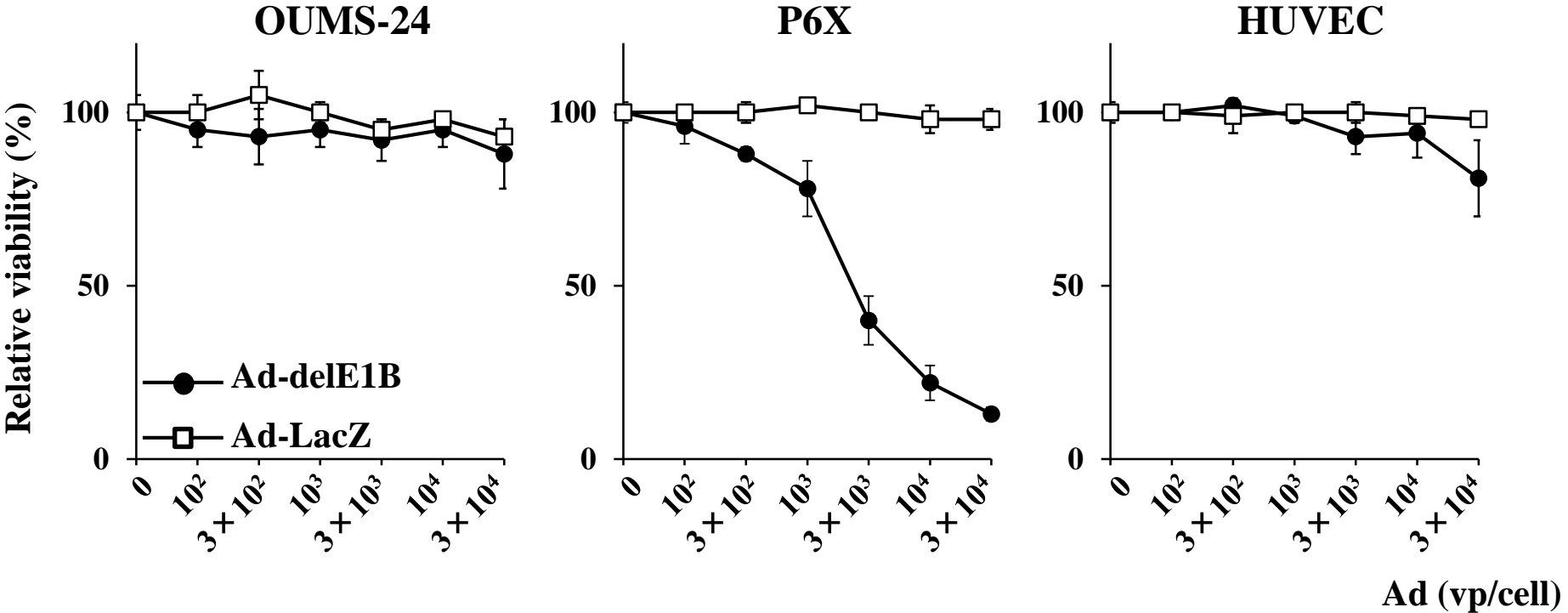

Supplement: Supplementary file 3 — Supplementary Figure 1 [file 41419_2021_3934_MOESM3_ESM.pdf]

Supplementary Figure 2

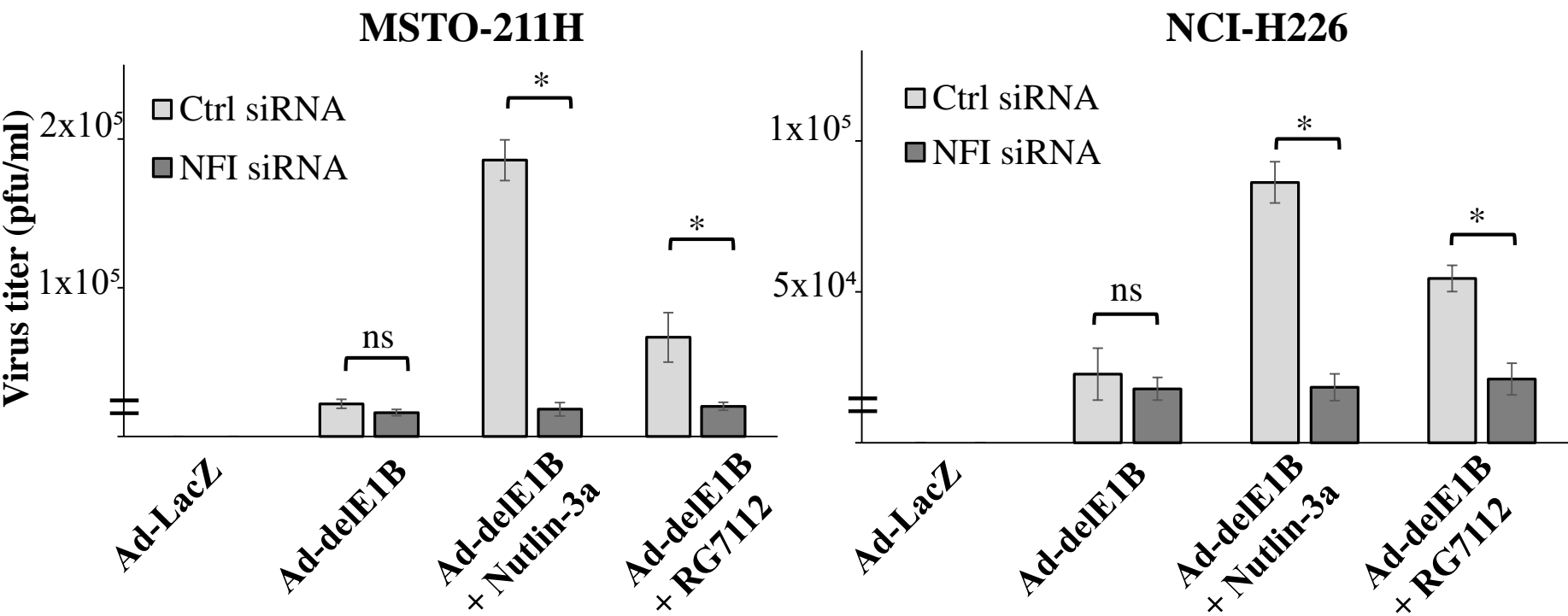

Supplement: Supplementary file 4 — Supplementary Figure 2 [file 41419_2021_3934_MOESM4_ESM.pdf]

Supplementary Figure 3

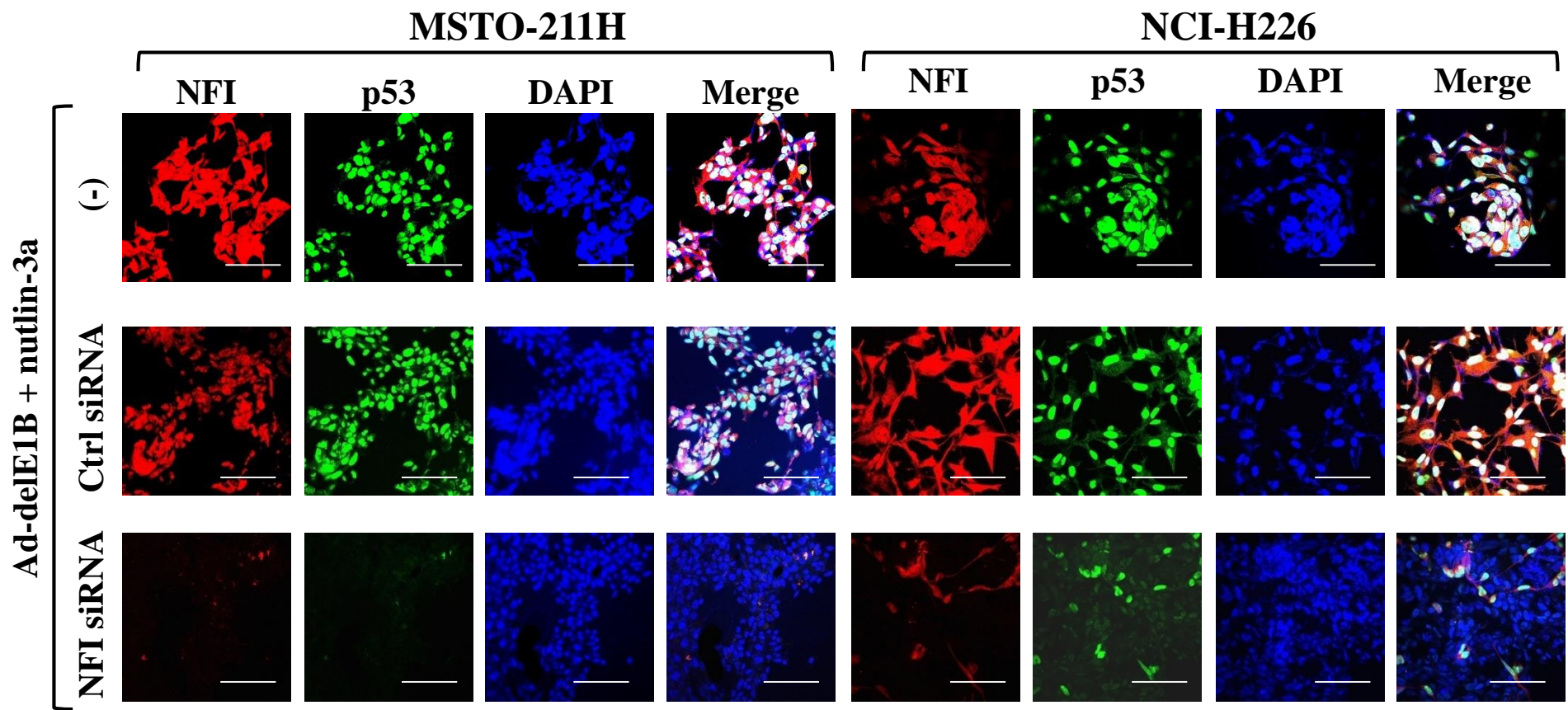

Supplement: Supplementary file 5 — Supplementary Figure 3 [file 41419_2021_3934_MOESM5_ESM.pdf]

## Slide 1
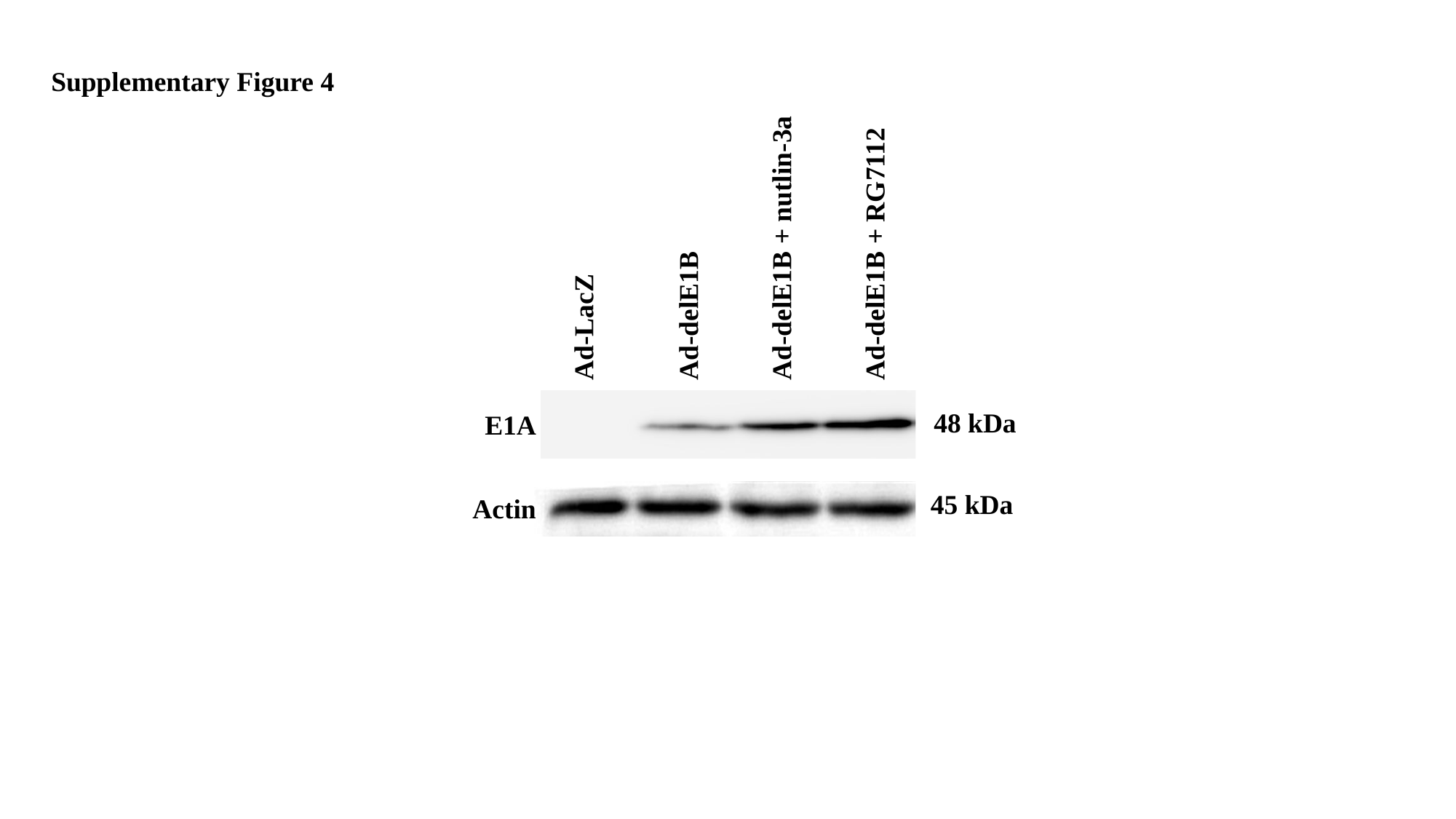

Supplementary Figure 4
Ad-delE1B + nutlin-3a
Ad-delE1B + RG7112
Ad-LacZ
Ad-delE1B
48 kDa
E1A
45 kDa
Actin

Supplement: Supplementary file 6 — Supplementary Figure 4 [file 41419_2021_3934_MOESM6_ESM.pptx]
